# 

**Published:** 1910-04

**Authors:** 


**The Propaganda for Reform in Proprietary Medicines.** Sixth Edition. Containing the various exposes of nostrums and quackery which have appeared in the Journal of the American Medical Association. Pp. 292. Illustrated. (Paper, 10 cents; cloth, 35 cents.)

The council on pharmacy and chemistry of the American Medical Association puts out annually this "propaganda." This is the edition for 1910.

---

**Handbook of Therapy.** Pp. 421. Chicago: American Medical Association, 1910. (Cloth, $1.50.)

### BOOKS RECEIVED.

---

International Clinics. A Quarterly of Illustrated Clinical Lectures and especially prepared articles on Treatment, Medicine, Surgery, Neurology, Pediatrics, Obstetrics, Gynecology, Orthopedics, Pathology, Dermatology, Ophthalmology, Otology, Rhinology, Laryngology, Hygiene, etc.—Edited by Henry W. Cattell, M. D. Vol. 1, twentieth series. Philadelphia and London: J. B. Lippincott Co. 1910. (Cloth, $2.00.)

Proceedings of the Third Annual Meeting of the Association of Life Insurance Presidents, held at Washington, D. C., January 19, and 20, 1910.

Proceedings of the National Confederation of State Medical Examining and Licensing Boards. Nineteenth Annual Convention held at Atlantic City, N. J., June 7, 1909. Murray Galt Motter, M.D., Secretary.

High Frequency Electric Currents in Medicine and Dentistry: Their Nature, Actions and Simplified Uses in External Treatments. By S. H. Monell, M.D. Illustrated. 8vo, 448 pages. Published by William R. Jenkins Co., 851-853 Sixth Avenue, New York. (Cloth, $4.00.)

Emergency Surgery. By John W. Sluss, M.D., professor of anatomy, Indiana University School of Medicine. Second edition. 12mo., pp. 762. Illustrated. Philadelphia: P. Blakiston's Son & Co. (Cloth, $3.50.)

Diseases of the Stomach and Intestines. By Robert Coleman Kemp, M.D., professor of gastrointestinal diseases in the New York School of Clinical Medicine. Octavo, pp. 766. Illustrated. Philadelphia and London: W. B. Saunders Co. (Cloth, $6.00.)

The Sexual Life of Woman in its Physiological, Pathological and Hygienic Aspects. By E. Heinrich Kisch, M.D., professor of the

German Faculty of the University of Prague. Translated by M. Eden Paul, M.D. Octavo, pp. 697. Illustrated. New York: Rebman Co. (Cloth, $5.00.)

Diseases of the Nose, Mouth, Pharynx and Larynx. By Alfred Bruck, Berlin. Translated by F. W. Forbes Ross, M.D., Edin., F.R.C.S., Eng. Octavo, pp. 639. Illustrated. New York: Rebman Co. (Cloth, $5.00.)

Principles of Surgery. By N. Senn, M.D., late professor of surgery, University of Chicago. Fourth edition by Emanuel J. Senn, M.D., and Emanuel Friend, M.D. Octavo, pp. 718. Illustrated. Philadelphia: F. A. Davis Co. (Cloth, $5.00.)

Essentials of Laboratory Diagnosis. By Francis Ashley Faught, M.D., director of the laboratory of clinical medicine, Medico-Chirurgical College, Philadelphia. 12 mo., pp. 317. Illustrated. Philadelphia: F. A. Davis Co. (Cloth, $2.00.)

The Examination of the Function of the Intestines by means of The Test Diet. By Professor Dr. Adolf Schmidt, Physician-in-Chief of the City Hospital, Friedrichstadt in Dresden. Translated by Charles D. Aaron, M.D., Professor of Diseases of the Stomach and Intestines in the Detroit Post-graduate School of Medicine. Second edition. 12 mo., pp. 133. Philadelphia: F. A. Davis Co. (Cloth, $1.00.)

Confessions of a Neurasthenic. By William Taylor Marrs, M.D. 12 mo., pp. 120. Philadelphia: F. A. Davis Co. (Cloth, $1.00.)

The Propaganda for Reform in Proprietary Medicines. Sixth Edition. Containing the various exposes of nostrums and quackery which have appeared in The Journal of the American Medical Association. Pp. 292. Illustrated. (Paper, 10 cents; cloth, 35 cents.)

Handbook of Therapy. Pp. 421. Chicago: American Medical Association, 1910. (Cloth, $1.50.)

New and Nonofficial Remedies. Containing descriptions of articles which have been accepted by the Council on Pharmacy and Chemistry of the American Medical Association, prior to January 1, 1910. (Paper, 25 cents; cloth, 50 cents.)

Progressive Medicine, Vol. 12, March, 1910. A Quarterly Digest of Advances, Discoveries and Improvements in the Medical and Surgical Sciences. Edited by Hobart Amory Hare, M.D., Professor of Therapeutics and Materia Medica in the Jefferson Medical College of Philadelphia. Lea & Febiger, Philadelphia and New York. (Per annum, paper bound, $6.00; cloth, $9.00.)

English Handbook to the Paris Medical School. By A. A. Warden, M.D., Paris. Second edition. Philadelphia: P. Blakiston's Son & Co. (Price, 50 cents.)

Twenty-sixth Annual Report of the State Board of Health, Michigan. For the year ending June 30, 1908. Frank W. Shumway, M.D., Secretary.

Annual Report of the Surgeon-General of the Marine Hospital Service of the United States, for the fiscal year 1909.

